# Pharmacological and rAAV Gene Therapy Rescue of Visual Functions in a Blind Mouse Model of Leber Congenital Amaurosis

**DOI:** 10.1371/journal.pmed.0020333

**Published:** 2005-11-01

**Authors:** Matthew L Batten, Yoshikazu Imanishi, Daniel C Tu, Thuy Doan, Li Zhu, Jijing Pang, Lyudmila Glushakova, Alexander R Moise, Wolfgang Baehr, Russell N. Van Gelder, William W Hauswirth, Fred Rieke, Krzysztof Palczewski

**Affiliations:** 1Department of Ophthalmology, University of Washington, Seattle, Washington, United States of America,; **2**Department of Pharmacology, Case School of Medicine, Case Western Reserve University, Cleveland, Ohio, United States of America,; **3**Department of Ophthalmology and Visual Sciences, Washington University School of Medicine, St. Louis, Missouri, United States of America,; **4**Department of Physiology and Biophysics, University of Washington, Seattle, Washington, United States of America,; **5**Department of Chemistry, University of Washington, Seattle, Washington, United States of America,; **6**Department of Ophthalmology, and Powell Gene Therapy Center, University of Florida, Gainesville, Florida, United States of America,; **7**Department of Ophthalmology, University of Utah, Salt Lake City, Utah, United States of America,; **8**Department of Biology, University of Utah, Salt Lake City, Utah, United States of America,; **9**Department of Neurobiology and Anatomy, University of Utah, Salt Lake City, Utah, United States of America,; **10**Department of Molecular Biology, Washington University School of Medicine, St. Louis, Missouri, United States of America,; **11**Department of Pharmacology, Washington University School of Medicine, St. Louis, Missouri, United States of America,; **12**Department of Pharmacology, University of Washington, Seattle, Washington, United States of America; Moorfields Eye HospitalUnited Kingdom

## Abstract

**Background:**

Leber congenital amaurosis (LCA), a heterogeneous early-onset retinal dystrophy, accounts for ~15% of inherited congenital blindness. One cause of LCA is loss of the enzyme lecithin:retinol acyl transferase (LRAT), which is required for regeneration of the visual photopigment in the retina.

**Methods and Findings:**

An animal model of LCA, the *Lrat* ^−/−^ mouse, recapitulates clinical features of the human disease. Here, we report that two interventions—intraocular gene therapy and oral pharmacologic treatment with novel retinoid compounds—each restore retinal function to *Lrat* ^−/−^ mice. Gene therapy using intraocular injection of recombinant adeno-associated virus carrying the *Lrat* gene successfully restored electroretinographic responses to ~50% of wild-type levels (*p* < 0.05 versus wild-type and knockout controls), and pupillary light responses (PLRs) of *Lrat* ^−/−^ mice increased ~2.5 log units (*p* < 0.05). Pharmacological intervention with orally administered pro-drugs 9-*cis*-retinyl acetate and 9-*cis*-retinyl succinate (which chemically bypass the LRAT-catalyzed step in chromophore regeneration) also caused long-lasting restoration of retinal function in LRAT-deficient mice and increased ERG response from ~5% of wild-type levels in *Lrat* ^−/−^ mice to ~50% of wild-type levels in treated *Lrat* ^−/−^ mice (*p* < 0.05 versus wild-type and knockout controls). The interventions produced markedly increased levels of visual pigment from undetectable levels to 600 pmoles per eye in retinoid treated mice, and ~1,000-fold improvements in PLR and electroretinogram sensitivity. The techniques were complementary when combined.

**Conclusion:**

Intraocular gene therapy and pharmacologic bypass provide highly effective and complementary means for restoring retinal function in this animal model of human hereditary blindness. These complementary methods offer hope of developing treatment to restore vision in humans with certain forms of hereditary congenital blindness.

## Abstract 2

A combination of intraocular gene therapy and pharmacologic bypass provides a complementary way to restore retinal function in an animal model of human hereditary blindness.

## Introduction

Development of successful treatments for inherited and acquired retinal disease caused by gene mutations represents a major challenge [1]. Recessive congenital defects arising from gene inactivation and subsequent disruption of a metabolic pathway are particularly amenable to pharmacological treatment or somatic gene therapy. Oral administration of appropriate compounds can correct visual deficits in humans and other animals by bypassing a block in the retinoid cycle [2]. Sorsby's fundus dystrophy and Leber congenital amaurosis (LCA) are two examples. Sorsby's fundus dystrophy, an autosomal-dominant retinal degeneration caused by mutations in the tissue inhibitor of the *metalloproteinases-3* gene, leads to night blindness [1]. Vitamin A (retinol [ROL]) administered orally has been shown to significantly restore photoreceptor function in affected individuals [3]. LCA is an early-onset recessive human retinal degeneration that can be caused by mutations in the gene encoding retinal pigment epithelium 65 (RPE65), a key protein involved in the production and recycling of 11-*cis*-retinal (11-*cis*-RAL) in the eye. Approximately 15% of patients with LCA have been found to have mutations in *Rpe65* [4,5]. Humans with this form of LCA and *Rpe65* ^−/−^ mice both have severely impaired rod and cone function [6]. The biochemical block caused by the absence of *Rpe65* ^−/−^ can be bypassed with synthetic *cis*-retinoids administered orally, resulting in a dramatic improvement in photoreceptor physiology [7].

Somatic gene therapy has also been very successful in many animal models of retinal degeneration [8]. Most notably, a canine model with a naturally occurring Rpe65 deficiency, the *Rpe65* ^−/−^ dog, bears a phenotype similar to that of human LCA patients and *Rpe65* ^−/−^ mice. A recombinant adeno-associated virus (rAAV) carrying wild-type (WT) *Rpe65* (rAAV-*Rpe65*) restored visual function in this model of childhood blindness [9].

The lecithin:retinol acyl transferase (LRAT)-deficient mouse is an animal model of LCA [10]. Mutations in the gene encoding LRAT are associated with early-onset severe retinal dystrophy, or LCA [11]. The prevalence of mutations at this locus among patients with LCA is unknown but it is likely uncommon, as three of 267 probands with severe early-onset retinal dystrophy carried mutations in *Lrat* [11]. LRAT is a key enzyme involved in storage of ROL in the form of retinyl esters (REs) in specific structures known as retinosomes [12]. Without LRAT, no 11-*cis*-RAL chromophore is produced, and visual function is severely impaired [10].

Here, we aimed to investigate whether we could rescue visual function by pharmacological intervention and gene transfer therapy in LRAT-deficient mice, and to assess the advantages and disadvantages of oral administration of retinoids and gene therapy. Comparison of these two approaches is an important prelude for treatment in humans.

## Methods

### Animals

All animal experiments employed procedures approved by the University of Washington and conformed to recommendations of the American Veterinary Medical Association Panel on Euthanasia and recommendations of the Association of Research for Vision and Ophthalmology. Animals were maintained in complete darkness, and all manipulations were done under dim red light employing a Kodak No. 1 safelight filter (transmittance > 560 nm). *Lrat* ^−/−^ mice were generated and genotyped as described previously [10]. Typically, 6- to 12-wk-old mice were used in all experiments. In the case of rAAV-*Lrat* treatment, 2- to 3-wk-old mice were used.

### rAAV1-VMD2-m*Lrat* Vector

The pTR-UF5 backbone [13] was used for generation of a pTR-VMD2-mLRAT plasmid construct. An EcoR1 fragment containing the full-length mouse *Lrat* cDNA was excised from pCR-TOPO II Blunt-*Lrat* and blunt ligated into the Not1 site of the UF5 cassette, replacing the *Gfp* gene. Orientation of the cDNA was confirmed by restriction analysis and by sequencing. The Kpn1-Xba1 fragment of the placF-VMD2 plasmid (from D. Zack, Johns Hopkins University) [14] includes the −585/+38 upstream region of the human VMD2 gene (Chromosome 11q13) and was subcloned into the Kpn1-Xba1 sites of the pTR-UF5 cassette (replacing the CMV promoter) upstream of mouse *Lrat* cDNA. Sequence analysis confirmed the orientation and reading frame of the *Lrat* cDNA. A serotype 1 AAV vector was produced in the presence of a mini-Ad helper plasmid pDG38 by double transfection of HEK293 cells, followed first by purification over an iodixanol gradient and then by high-Q FPLC column chromatography (Pharmacia, Uppsala, Sweden). Vector particle titers were determined by quantitative PCR. The rAAV1-VMD2-m*Lrat* vector was prepared at 4 × 10^13^ physical particles/ml. Exclusive RPE expression is seen in mice using a *Gfp* reporter gene in an analogous rAAV1-VMD2 vector (Glushakova and Hauswirth, unpublished data).

### Preparation of Retinoids and Oral Gavage

All*-trans-*retinyl acetate (R-Ac), all*-trans-*R-Palm, all*-trans-*RAL, all*-trans-*ROL, and 9*-cis-*RAL were purchased from Sigma-Aldrich (St. Louis, Missouri, United States). 9*-cis-*ROL, 9*-cis-*R-Palm, 9*-cis-*R-Ac, and 9*-cis-*retinyl succinate (9*-cis-*R-Su) were prepared from 9*-cis-*RAL. To prepare 9*-cis-*R-Ac, 100 mg of 9*-cis-*RAL was reduced with 50 mg of sodium borohydride in 0.7 ml of ethanol at 0 °C for 30 min, and 9*-cis-*ROL was purified by organic extraction and dried under argon. Solid 9*-cis-*ROL and 80 mg of 4-dimethylaminopyridine were dissolved in 0.4 ml of dry CH_2_Cl_2,_ and 0.1 ml of acetic acid anhydride was added. After 6 h at 10 °C, the reaction was quenched with 0.1 ml of ethanol, CH_2_Cl_2_ was removed by flowing argon at 20 °C, and 9*-cis-*R-Ac was purified by organic extraction and dried under argon. To prepare all*-trans-*R-Su or 9*-cis-*R-Su, solid all*-trans-*ROL or 9*-cis-*ROL was dissolved in 0.2 ml of pyridine, with 100 mg of succinic acid anhydride added and reacted overnight at 10 °C. CH_2_Cl_2_ was removed by flowing argon at 20 °C, and all*-trans-*R-Su or 9*-cis-*R-Su was then purified by organic extraction and dried down under argon. Retinoids were dissolved in pure canola oil (Western Family Foods, Tigard, Oregon, United States), and concentrations were measured spectrophotometrically. Retinoids at a final concentration of 40 mg/ml in canola oil were administered to *Lrat* ^−/−^ mice using a 1-ml syringe and a 20-gauge, 3.5-cm long gavage needle. Mice were allowed to rest for 3 d following gavage. A single 5-mg dose (125 μl) of retinoid was used to compare the different retinoids. Doses of gavaged 9*-cis-*R-Ac were 1, 2, 5, 10, 20, and 40 μmol to determine dose effect. Multiple gavages of 9-*cis-*R-Ac were performed with doses of 1, 5, and 10 μmol for up to ten consecutive treatments.

### Photobleaching of 9-*cis*-R-Ac Gavaged Mice

*Lrat* ^−/−^ mice were gavaged four times with 10 μmol doses of 9-*cis*-R-Ac. Caged mice were placed on a bench under fluorescent light of average luminosity 600 cd × m^−2^ and allowed to photobleach for 1, 3, and 10 d. Mice were then placed in the dark for 1 d, sacrificed, and the eyes collected for retinoid analysis. A subset of mice were gavaged with 10 μmol 9-*cis*-R-Ac following photobleach, placed in the dark for 1 d, sacrificed, and the eyes collected for retinoid analysis.

### Pupillary Light Responses

Pupillary light responses (PLRs) were recorded from dark-adapted mice under infrared conditions using a CCD video camera fitted with close-up lens and an IR filter. Data analysis was performed by video pupillometry. Light stimuli were provided by a halogen source; wavelength and intensity were manipulated with neutral density and narrow bandwidth (10 nm) interference filters (Oriel, Stratford, Connecticut, United States). Irradiance measurements (W/m^2^) were made using a radiometer (Advanced Photonics International, White Plains, New York, United States).

### Analyses of Retinoids and Visual Pigments

All procedures were performed under dim red light as described previously [7,15,16]. Retinoid analysis was performed on an Agilent 1100 series HPLC equipped with a diode array detector and Agilent Chemstation A.10.01 software (Agilent, Palo Alto, California, United States). A normal phase column (Beckman Ultrasphere Si 5μ, 4.6 × 250 mm [Beckman Instruments, Fullerton, California, United States]) and an isocratic solvent system of 0.5% ethyl acetate in hexane (v/v) for 15 min followed by 4% ethyl acetate in hexane for 65 min at a flow rate of 1.4 ml/min at 20 °C (total 80 min) with detection at 325 nm were used.

All of the experimental procedures related to the analysis of dissected mouse eyes, derivatization, and separation of retinoids have been described previously [7]. Rhodopsin and isorhodopsin measurements were performed as described previously [17]. Typically, two mouse eyes were used per assay, and the assays were repeated three to six times. Whole livers were homogenized for 30 s in 4 ml of retinoid derivatization buffer (50 mM MOPS, 10 mM NH_2_OH, and 50% ethanol in H_2_O [pH 7.0]) using a Polytron PT1200 motorized homogenizer (Polytron, Bad Wildbad, Germany), and allowed to sit at room temperature for 30 min. Retinoid analysis was performed on 1 ml of liver homogenate following the same extraction procedure used for eyes. Mouse blood was collected from the eye socket using heparinized microhematocrit capillary tubes (Fisher Scientific) immediately following removal of the eyes. Blood was transferred to a tared 1.5-ml Eppendorf tube and weighed (Eppendorf, Hamburg, Germany). Next, 1 ml of retinoid derivatization buffer was added and vortexed (Eppendorf Mixer 5432) for 30 min high speed at room temperature. Blood was then used for retinoid analysis following the same extraction procedure used for eyes. Rhodopsin from *Lrat* ^−/−^ mice treated with rAAV-*Lrat* virus was isolated by immunoaffinity chromatography as described previously [18].

### Electroretinography

Mice were anesthetized by intraperitoneal injection using 20 μl/g body weight of 6 mg/ml ketamine and 0.44 mg/ml xylazine diluted with 10 mM sodium phosphate (pH 7.2), containing 100 mM NaCl. The pupils were dilated with 1% tropicamide. A contact lens electrode was placed on the eye, and ground electrodes were placed on the scalp and tail. Electroretinograms (ERGs) were recorded with the universal testing and electrophysiologic system UTAS E-3000 (LKC Technologies, Gaithersburg, Maryland, United States). The light intensity was calibrated and computer-controlled. The mice were placed in a Ganzfeld chamber, and scotopic responses to flash stimuli were obtained from both eyes simultaneously. Flash stimuli had a range of intensities (−3.7 to 2.8 log cd·s·m^−2^), and white light flash duration was adjusted according to intensity (from 20 μs to 1 ms). Three to five recordings were made with intervals of 10 s or longer, and for higher intensity trials, intervals were 10 min or as indicated. Five animals were typically used for recording of each point in gavage conditions. ERGs were performed on all mice treated with rAAV-*Lrat*. The results were examined using the one-way ANOVA test.

### Recordings from Rods

Suction electrode recordings from rod photoreceptors followed published procedures [19]. In brief, a small piece of retina was shredded with fine needles, and the resulting suspension was placed in a chamber on the microscope stage. Single outer segments were drawn by suction into a tightly fitting glass electrode, and changes in outer segment current in response to brief light flashes were measured. All procedures were carried out using infrared illumination (>950 nm). Mice for these experiments were dark adapted for at least 12 h. C57Bl/6J mice were used as controls. All experiments were carried out at 35–37 °C.

### Liver RA Analysis

The method of Kane et al. was used with slight variation [20]. Whole mouse livers were removed from *Lrat* ^−/−^ mice after eye removal during the above experiments. Livers were weighed and then frozen in liquid N_2_. Frozen livers were transferred to a 15-ml glass centrifuge tube (Corex #8441 [Corning Life Sciences, Acton, Massachusetts, United States]) containing ice-cold phosphate buffered saline (PBS) at a 1:4 ratio of liver to PBS (w/v) to make a 25% homogenate and were homogenized 30 s using a Polytron PT1200 motorized homogenizer. Next, 500 μl of homogenate was transferred to an 8-ml glass tube on ice, and 1 ml of ice-cold ethanol and 5 μl of 5 M NaOH were added and vortexed. Finally, 4 ml of ice-cold hexane was added, and the mixture was vortexed and centrifuged for 5 min using a Beckman J2-HS centrifuge with a JS13.1 swinging bucket rotor for 5 min at 4,000 rpm, 4 °C. The hexane layer was removed and discarded. The hexane extraction was repeated one more time. To extract RA, 20 μl of 12 M HCl was added to the remaining aqueous solution and vortexed. Hexane extraction was performed as above, but hexane was retained from both extractions and dried down under blowing argon at 20 °C. Residue was dissolved in 300 μl of 1,000:4.3:0.675 hexane:2-propanol:acetic acid (v/v) and transferred to an amber glass HPLC vial with glass insert.

For the first separation, 100 μl of sample was injected into the Agilent 1100 HPLC described above. Two tandem normal phase columns were used. The first column was a Varian Microsorb Silica 3μ, 4.6 × 100 mm column (Varian, Palo Alto, California, United States), and the second was the Beckman column described above. An isocratic solvent system of 1,000:4.3:0.675 hexane:2-propanol:glacial acetic acid (v/v) at a flow rate of 1 ml/min at 20 °C with detection at 355 nm was used [21]. The system was calibrated using standards of all-*trans*-RA and 9-*cis*-RA purchased from Sigma-Aldrich.

### Immunocytochemistry and Histology

Procedures have been described previously [22]. Anti-LRAT monoclonal antibody [10] was directly coupled with Alexa488, or detected by anti-mouse IgG labeled with Cy3. Sections were analyzed under an epifluorescence microscope (Nikon, Tokyo, Japan). Low magnification images were captured with a digital camera (ORCA-ER, Hamamatsu Photonics, Bridgewater, New Jersey, United States) or a Zeiss LSM 510 NLO confocal microscope (Zeiss, Oberkochen, Germany).

Retinas of 17-mo-old *Lrat* ^−/−^ and WT mice were marked, enucleated, and immersed immediately in a fixative of 4% paraformaldehyde in 0.1 M phosphate buffer (pH 7.4). Following fixation for 4–5 h at 4 °C, the eyecup containing the optic nerve was postfixed in 1% osmium tetroxide in phosphate buffer, dehydrated through a series of graded ethanol, and embedded in Spurr's resin. Sections 0.5–1 μm thick were imaged using a Leica (Wetzlar, Germany) DM-R microscope with Prior stage, using Syncroscan RT software from Syncroscopy (Frederick, Maryland, United States). The scaling for measurement is 182 nm/pixel and 5.5 pixels/micrometer at 40×. The thicknesses of rod outer segments (ROSs) in micrometers as a function of distance from the ONH were measured after import into Deneba Canvas software (ACD Systems, Saanichton, British Columbia, Canada).

### Transmission EM

For transmission electron microscopy (EM), mouse eyecups were fixed primarily by immersion in 2.5% glutaraldehyde and 1.6% paraformaldehyde in 0.08 M PIPES (pH 7.4) containing 2% sucrose, initially at room temperature for ~1 h, then at 4 °C for the remainder of 24 h. The eyecups were then washed with 0.13 M sodium phosphate (pH 7.3), and secondarily fixed with 1% OsO_4_ in 0.1 M sodium phosphate (pH 7.4), for 1 h at room temperature. The eyecups were dehydrated through a CH_3_OH series and transitioned to the epoxy embedding medium with propylene oxide. The eyecups were embedded for sectioning in Eponate 812. Ultrathin sections (60–70 nm) were stained with aqueous saturated uranium acetate and Reynold's formula lead citrate prior to survey and micrography with a Philips CM10 EM (Philips Electron Optics, Eindhoven, The Netherlands).

### Statistical Analyses

Data were expressed as mean ± standard error of the mean (SEM) Liver data was presented as mean ± SEM of one-quarter of whole livers. Blood data was presented as mean ± SEM per 100 mg of blood.

## Results

### *Lrat* ^−/−^ Mice Are a Model of Vitamin A Deficiency

We analyzed the retinoid content of *Lrat* ^−/−^ and *Lrat* ^+/+^ mice to determine how the absence of LRAT influenced the levels of the 11-*cis*-RAL chromophore and its derivatives. *Lrat* ^−/−^ mice had diminished levels of all-*trans*-retinoids in the eye [10] and 100- to 1,000-fold lower levels of *cis*-retinoids compared with WT mice (Figure S1). *Cis*-retinoids were also observed in *Rpe65*-deficient mice, but at 5% of the level of WT mice [23]. Formation of *cis*-retinoids is likely due to the propensity of retinoids to spontaneously isomerize and become trapped by opsin in the photoreceptors. *Lrat* is expressed in the liver as well as in the eye; the amount of REs in the liver of *Lrat* ^−/−^ mice was more than 20,000-fold lower than WT mice (Table 1). Circulating all-*trans*-ROL from the diet was also reduced, producing lower retinoid concentrations in the blood (Table 1). No visual pigments were measurable in the *Lrat* ^−/−^ mice by direct spectrophotometric analysis.

**Table 1:**
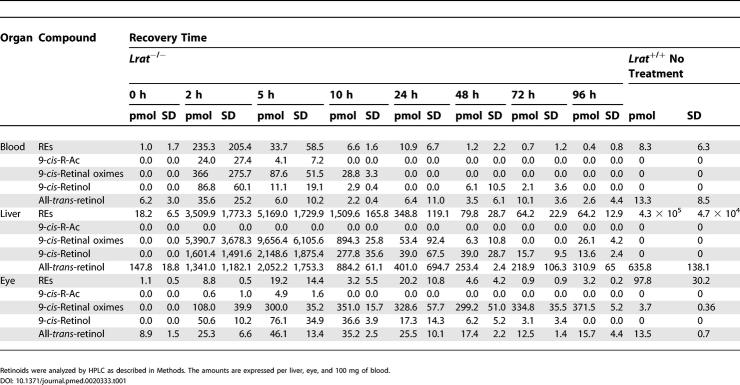
Retinoid Analysis of Tissues from *Lrat* ^−/−^ Mice Gavaged with 20 μmol 9-*cis*-R-Ac as a Function of Post-Treatment Time

### Visual Pigment in *Lrat*-Deficient Mice Is Restored by Oral Gavage with 9-*cis*-Retinoid

**Figure 1:**
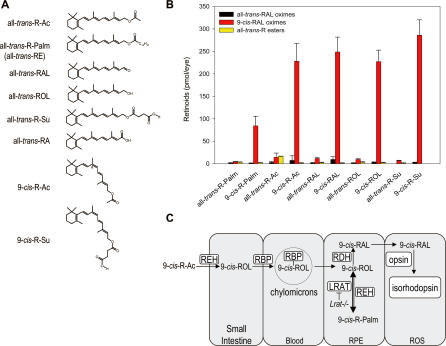
Retinoid Structures, Specificity of Retinoids in Regeneration of Visual Pigment, and Model of Absorption of 9-*cis*-R-Ac in Mammals (A) Structures of retinoids used for gavage studies. (B) Levels of all-*trans*-RAL oximes, 9-*cis*-RAL oximes (corresponding to formation of visual pigment isorhodopsin), and all-*trans*-REs in *Lrat* ^−/−^ mouse eyes gavaged with 5 mg of each retinoid before 48–72 h dark adaptation (*n* ≥ 3, data shown with standard deviation [SD]). (C) Model of absorption of 9-*cis*-R-Ac in mammals.

We synthesized a series of 9-*cis*-retinoids and ester derivatives to determine whether the phenotype of *Lrat* ^−/−^ mice could be reversed by chemically bypassing the LRAT-catalyzed metabolic step (Figure 1A). Oral gavage of these exogenous retinoids led to the generation of visual pigment in *Lrat* ^−/−^ mice. Visual pigments were measured chromatographically by the retention of 9-*cis*-RAL oximes (Figure 1B) or spectrophotometrically (unpublished data) by the level of isorhodopsin (i.e., opsin + 9-*cis*-RAL chromophore). Both methods were highly reproducible and yielded similar results. Visual pigment was rescued in *Lrat* ^−/−^ mice by oral gavage with 9-*cis*-RE, 9-*cis*-RAL, or 9-*cis*-ROL, whereas all-*trans* isomers were ineffective. Among REs, 9-*cis*-R-Ac and 9-*cis*-R-Su were most efficient (by weight per dose) in restoring pigment (Figure 1B). Esters are readily metabolized in the small intestine and are more inert than RALs and ROLs (Figure 1C). For these reasons 9-*cis*-R-Ac was chosen for the remaining experiments.

### Kinetics of Visual Pigment Rescue and Retinoid Clearance by Oral 9-*cis*-R-Ac in *Lrat*-Deficient Mice

**Figure 2:**
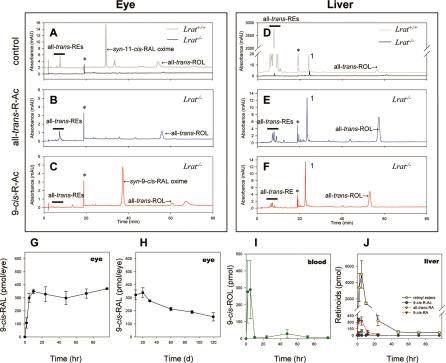
Retinoids in the Liver and Eyes of *Lrat* ^−/−^ Mice after 9-*cis*-R-Ac Treatment (A–F) Normal-phase HPLC analysis of nonpolar retinoids extracted from the tissues of dark-adapted *Lrat* ^+/+^ or *Lrat* ^−/−^ mice following gavage with retinoids. Peaks marked * represent the solvent change artifact, and peaks labeled (1) indicate an unidentified non-specific compound with λ_max_= 270 nm. In all experiments, mice were dark-adapted for 48 h after gavage. Shown are results from eyes of dark-adapted control *Lrat* ^+/+^ (gray) and *Lrat* ^−/−^ mice (black) (A); eyes of dark-adapted *Lrat* ^−/−^ mouse gavaged with all-*trans*-R-Ac (B); eyes of dark-adapted *Lrat* ^−/−^ mouse gavaged with 9-*cis*-R-Ac (C); liver tissue from dark-adapted control *Lrat* ^+/+^ (gray) and *Lrat* ^−/−^ (black) mice (D); liver tissue from dark-adapted *Lrat* ^−/−^ mouse gavaged with all-*trans*-R-Ac (E); and liver tissue from dark-adapted *Lrat* ^−/−^ mouse gavaged with 9-*cis*-R-Ac (F). (G–J) Time course of the levels of nonpolar and polar retinoids in the tissues of *Lrat* ^−/−^ mice following gavage with 9-*cis*-R-Ac measured by HPLC. After gavage, mice were dark adapted for indicated time before HPLC analysis (*n* ≥ 3, data shown with SD). The graphs depict: a short time course of 9-*cis*-RAL oxime levels detected in *Lrat* ^−/−^ mice eyes following a 20 μmol gavage of 9-*cis*-R-Ac (G); a longer time course of 9-*cis*-RAL oxime levels in *Lrat* ^−/−^ mice eyes following a 20 μmol gavage of 9-*cis*-R-Ac (H); time course of 9-*cis*-ROL blood levels in *Lrat* ^−/−^ mice following gavage with 20 μmol 9-*cis*-R-Ac (I); and time course of RE and RA levels in liver of *Lrat* ^−/−^ mice following a 20 μmol gavage with 9-*cis*-R-Ac (J).

Gavage with 9-*cis*-R-Ac produced a transient increase in retinoid levels in the liver and a more sustained increase in levels in the eye (Figure 2). The visual pigment was restored in *Lrat* ^−/−^ mice 4–5 h after gavage with 9-*cis*-R-Ac. Visual pigment levels measured by HPLC remained nearly constant for 96 h (Figure 2G). By 120 d following a single oral gavage with 9-*cis*-R-Ac, dark-reared *Lrat* ^−/−^ mice retained more than 50% of the pigment (Figure 2H). Considering that~10% of ROSs are phagocytosed daily and replaced by newly formed discs at their base [24], these data suggest that even in the absence of LRAT, the chromophore may be efficiently recycled via hydrolysis of isorhodopsin and directional transport of 9-*cis*-RAL from the RPE to the photoreceptors.

Despite the absence of LRAT, REs were formed transiently in the liver and blood after gavage, suggesting that another enzyme is capable of the esterification reaction, such as acyl coenzyme A:diacylglycerol acyltransferase [25]. In less than 10 h, more than 90% of retinoids, including nuclear receptor activators all-*trans*-RA and 9-*cis*-RA, were cleared (Figure 2I and 2J; Table 1). Thus within 4–5 h after gavage, peripheral hydrolysis of REs is faster than synthesis, and retinoids are quickly metabolized or secreted. These data suggest efficient uptake of 9-*cis*-retinoids by the visual pigment and rapid clearance of excess exogenous retinoids from key metabolizing and transporting tissues, lowering the potential for toxicity.

### Regeneration of Visual Pigments Improves the Morphology of Rod Photoreceptors

**Figure 3:**
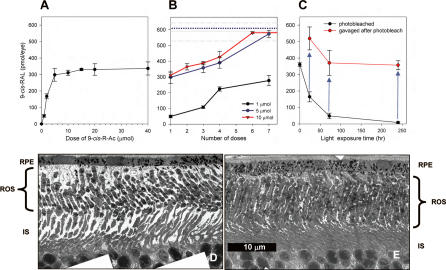
Levels of 9-*cis*-RAL Oximes in the Eyes of *Lrat* ^−/−^ Mice after a Single or Multiple Dose of 9-*cis*-R-Ac (A) The level of 9-*cis*-RAL in *Lrat* ^−/−^ mouse eyes after a varying dose of 9-*cis*-R-Ac. (B) The level of 9-*cis*-RAL in *Lrat* ^−/−^ mouse eyes after a varying size and number of doses of 9-*cis*-R-Ac. The solid gray line represents a maximal level of isorhodopsin as measured by the level of 9-*cis*-retinal oximes in *Lrat* ^−/−^ mouse eyes after ten gavages; dashed gray line indicates the SD. The maximal level of isorhodopsin is comparable to the level of rhodopsin in WT mice (blue dotted line, shown as pmol of 11-*cis*-retinal/eye). (C) The level of 9-*cis*-RAL in *Lrat* ^−/−^ mouse eyes after 9-*cis*-R-Ac treatment and light exposure or after exposure to light and re-gavage (*n* ≥ 3, data shown with SD). (D and E) Changes in the RPE-ROS interface in control *Lrat* ^−/−^ mice and *Lrat* ^−/−^ mice treated with 9-*cis-*R-Ac. Treated *Lrat* ^−/−^ mice were gavaged with 9-*cis*-R-Ac (10 μmol per gavage) six times, 3 d apart, and analyzed (D). Control retina from age-matched (8 wk old) untreated *Lrat* ^−/−^ mice (E). Considerably improved RPE-ROS processes were observed in all treated mice. RPE, retinal pigment epithelium; ROS, rod outer segments; IS, inner segments. Scale bar, 10 μm.

Maximal visual pigment formation required ~4 μmol of 9-*cis*-R-Ac (Figure 3A). The level of isorhodopsin in the eye of *Lrat* ^−/−^ mice after a single gavage was ~60%–70% of the WT level of rhodopsin (Figure 3). When total opsin was isolated by immunoaffinity chromatography, the UV-vis spectrum showed that all opsin present in the retina was regenerated (unpublished data), suggesting that all opsin was properly folded but is present in ROS at lower amounts than in WT ROS. Multiple 5- to 10-μmol doses spread over weeks produced almost full recovery of WT levels of visual pigment (Figure 3B).

Exposure to light released the isomerized chromophore as all-*trans*-ROL in treated *Lrat*-deficient mice (Figure 3C, black) with kinetics proportional to the intensity of the bleaching light. Subsequent gavages restored the chromophore efficiently (Figure 3C, red). Multiple gavages were effective in mice up to 12 mo old (Figure S2A). Histological analysis showed that the retina of untreated *Lrat* ^−/−^ mice degenerated slowly (Figure S2B), leaving functionally intact photoreceptors available for treatment in the older mice.

Since ROS structural morphology is thought to be dependent on functional rhodopsin, this result (in conjunction with recycling of the chromophore as discussed above) suggested that ROS morphology might be improved with treatment. We used EM to evaluate the RPE-ROS interface. Control and gavaged *Lrat*-deficient mice were analyzed. The thickness of the ROS layer was substantially improved, from 10.7 ± 0.16 μm to 14.2 ± 2.2 μm in treated mice, and the RPE-ROS interface showed closer apposition in comparable areas of the retina (Figure 3D and 3E; *n* = 5, *p* < 0.002).

### Improvement of Rod Responses in *Lrat* ^−/−^ Mice Treated with 9-*cis*-R-Ac

**Figure 4:**
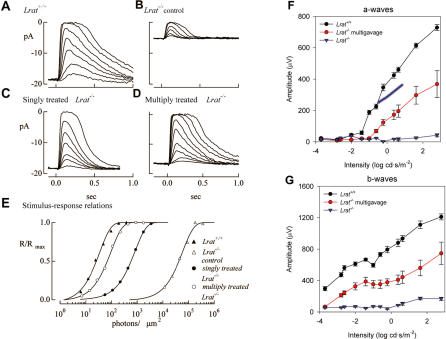
Rescue of Visual Responses Measured by Single-Cell Recording and ERG Responses of single *Lrat* ^+/+^ and *Lrat* ^−/−^ Rods (A–D) Flash families measured for a *Lrat* ^+/+^ rod (A), a control *Lrat* ^−/−^ rod (B), a *Lrat* ^−/−^ rod after a single gavage with 9-*cis*-R-Ac (C), and a *Lrat* ^−/−^ rod after three gavages with 9-*cis*-R-Ac (D). Each panel superimposes average responses to five to 20 repeats of a flash, with the flash strength increasing by a factor of two for each successively larger response. (E) Stimulus-response relations for the same cells in (A–D). Maximal response amplitudes are plotted against the flash strength. Fits are saturating exponential functions, used to estimate the strength of the flash producing a half-maximal response (*Lrat* ^+/+^, 26 photons/μm^2^; *Lrat* ^−/−^, 43,000 photons/μm^2^; singly treated *Lrat* ^−/−^, 590 photons/μm^2^; and multiply treated *Lrat* ^−/−^, 69 photons/μm^2^). (F and G) Comparison of WT mice scotopic single flash ERG to *Lrat* ^−/−^ 9-*cis*-R-Ac gavaged mice and *Lrat* ^−/−^ and *Lrat* ^+/+^ control mice. *Lrat* ^−/−^ mice were gavaged nine times with 5 μmol 9-*cis*-R-Ac over a 1-mo time period (*n* ≥ 3, data shown with SD).

Outer segment membrane currents of *Lrat* ^+/+^ and *Lrat* ^−/−^ rods were directly measured with suction electrodes (Figure 4). Rods of untreated *Lrat* ^−/−^ mice were ~2,000-fold less sensitive than *Lrat* ^+/+^ rods. Gavage of *Lrat* ^−/−^ mice with 9-*cis*-R-Ac restored near-WT sensitivity, although several treatments were required.

Figure 4A and 4B show flash families recorded from *Lrat* ^+/+^ and *Lrat* ^−/−^ rods. *Lrat* ^−/−^ rods had a smaller dark current than *Lrat* ^+/+^ rods (mean ± SEM: *Lrat* ^−/−^, 4.8 ± 0.4 pA, *n* = 21; *Lrat* ^+/+^, 15 ± 1 pA, *n* = 22). In addition, the dim flash response reached a peak more quickly in *Lrat* ^−/−^ rods (*Lrat* ^−/−^, 108 ± 4 msec; *Lrat* ^+/+^, 228 ± 9 msec). Sensitivity was estimated by plotting the amplitude of the response versus flash strength (Figure 4E). The flash necessary to produce a half-maximal response was ~2,000 times brighter in *Lrat* ^−/−^ rods (*Lrat* ^−/−^, 33,000 ± 2,000 photons/μm^2^; *Lrat* ^+/+^, 18 ± 1 photons/μm^2^). The decreased dark current, decreased sensitivity, and faster response kinetics of *Lrat* ^−/−^ rods were all consistent with bleaching adaptation produced by residual opsin activity in the absence of a chromophore [26–28]. This phenotype is more severe than that of *Rpe65* ^−/−^ rods, which are ~150-fold less sensitive than normal [16]. This may be explained by the lower levels of 9-*cis*-RAL (see Figure S1) [23] seen in *Lrat* ^−/−^ mice compared with *Rpe65* ^−/−^ mice. The sensitivity of *Lrat* ^−/−^ rods was markedly enhanced when the mice were gavaged with 9-*cis*-R-Ac. Figure 4C and 4D show flash families recorded from *Lrat* ^−/−^ mice rods after a single gavage (Figure 4C) and multiple gavages (Figure 4D). Figure 4E shows the corresponding stimulus-response relations. The dark current was restored to near-normal levels after a single treatment with 9-*cis*-R-Ac (15 ± 1 pA, *n* = 10), and the half-maximal flash strength was reduced to 390 ± 20 photons/μm^2^ (*n* = 10). Correcting for the ~3-fold reduced quantum efficiency from rhodopsin to isorhodopsin, the sensitivity of singly-treated rods was ~8-fold less than normal. Treatment with multiple doses of 9-*cis*-R-Ac increased the sensitivity. The half-maximal flash strength after three treatments was 87 ± 6 photons/μm^2^ (*n* = 26). Correcting for the lowered 9-*cis*-RAL quantum efficiency, this is within a factor of two of WT mouse rod responses.

### Restoration of ERG in *Lrat* ^−/−^ Mice Treated with 9-*cis*-R-Ac

ERG responses in treated and control *Lrat* ^−/−^ mice confirmed the improvements described above for individual rod signaling. The a-wave (generated by photoreceptors) and b-wave (generated by bipolar cells) of dark-adapted *Lrat* ^−/−^ mice were about 5% of the amplitude of those observed in WT mice (Figure 4F). The *Lrat* ^−/−^ b-wave was also reduced substantially, in proportion to the decreased a-wave. Following multiple 9-*cis*-R-Ac gavage treatment, ERG a- and b-wave amplitudes increased to about half of WT levels (Figure 4F and 4G).

### AAV-*Lrat* Rescue of Visual Function in *Lrat*-Deficient Mice

**Figure 5:**
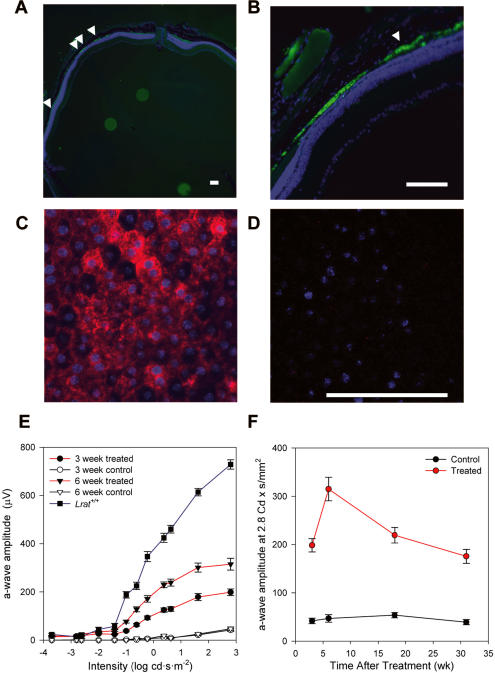
Immunocytochemistry and ERG of rAAV-*Lrat*-Treated *Lrat* ^−/−^ Mice (A) Immunolocalization of LRAT (green) in rAAV-*Lrat* treated *Lrat* ^−/−^ mouse eye. Anti-LRAT antibody was directly labeled by Alexa 488. Expression of LRAT is locally restricted (arrowheads) in the eye. Nuclei are stained by Hoechst 33342 (blue). (B) Higher-power magnification image of (A). LRAT (green) is observed specifically in the RPE cell layer. (C) Subcellular localization of LRAT in rAAV-*Lrat* treated *Lrat* ^−/−^ mouse eye. RPE cells were labeled by anti-LRAT and detected by Cy3-labeled secondary antibody (red). Nuclei are stained by Hoechst 33342 (blue). RPE cell layer was mounted flat on a coverslip and imaged. LRAT is localized in the internal membrane of the RPE cells. Similar localization was observed for WT mouse RPE cells [12]. (D) Control flat-mounted RPE cell layer of untreated *Lrat* ^−/−^ mouse. Anti-LRAT antibody does not show any non-specific labeling. Bars indicate 100 μm. (E) Comparison of scotopic single flash ERG of rAAV-*Lrat* treated, *Lrat* ^+/+^, *and Lrat* ^−/−^ control mice as measured by a-wave amplitudes, (*n* ≥ 16, data shown with SD). (F) A plot of a-wave amplitudes at 2.8 cd·s·m^−2^ intensity as a function of post-treatment time for rAAV-*Lrat* treated mice.

As an alternative to oral gavage, we tested the ability of treatment with rAAV carrying the *Lrat* gene to restore function in *Lrat*-deficient mice. Immunolocalization of LRAT in rAAV-*Lrat*-treated *Lrat* ^−/−^ mouse eyes showed expression in the vicinity of the injection site (Figure 5A) and was not uniform (Figure 5B). Higher resolution flat-mounted immunocytochemistry showed that *Lrat* was expressed specifically in the RPE of treated mice, but was not observed in control mice (Figure 5C and 5D). Treatment led to production of rhodopsin as determined by retinoid analysis and isolation of rhodopsin (Figure S3). The measurements of the level of rhodopsin as measured both directly by spectroscopy and indirectly by *cis*-retinoids (11- or 9-) are in good agreement (see also [29]).

Treatment with rAAV-*Lrat* increased the sensitivity of the a- and b-waves in *Lrat* ^−/−^ mice (Figure 5E and 5F). The rescue was comparable to 9-*cis*-R-Ac-treated mice. The largest improvement in sensitivity was seen 6 wk after injection of the virus, while the response gradually declined from 6–31 wk post-treatment (Figure 5F). The improvement in a- and b-wave sensitivity following rAAV-*Lrat* treatment showed considerable variability, most likely due to the inherent variability in the amount of vector surgically delivered to these small eyes (Figure S4).

The visual pigment of rAAV-*Lrat*-treated *Lrat-*deficient mice was augmented by oral gavage with 9-*cis*-R-Ac as described above. This augmentation was observed by HPLC separation and quantification of 11-*cis*-RAL oximes (a result of rAAV rescue) and 9-*cis*-RAL oximes (Figure S3A–S3C). ERG responses in *Lrat* ^−/−^ mice treated with rAAV-*Lrat* and 9-*cis*-R-Ac further improved to reach the levels of 9-*cis*-R-Ac treated mice (Figure S3D and S3E).

### Both rAAV-*Lrat* and 9-*cis*-R-Ac Treatment Restore PLRs in *Lrat*-Deficient Mice

**Figure 6:**
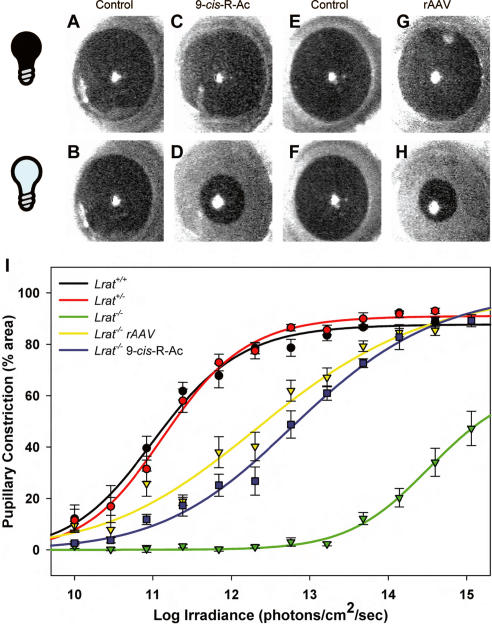
Light-Induced Pupillary Constriction of *Lrat* ^−/−^ Mice Before and After Treatment with 9-*cis*-R-Ac or rAAV-*Lrat* (A–H) 470 nm light (4.79 × 10^13^ photons·cm^−2^·sec^−1^) was used to stimulate pupillary constriction. Untreated *Lrat* ^−/−^ pupil before (A) and during (B) light exposure. Same mouse as in (A and B) subsequent to treatment with 9-*cis*-R-Ac, before (C) and during (D) light exposure. Control, untreated pupil of *Lrat* ^−/−^ mouse before (E) and during (F) light exposure. Contralateral eye of mouse shown in (E and F) treated with rAAV-*Lrat*, before (G) and during (H) light exposure. (I) Irradiance-response relations for PLR to 470 nm light.

PLRs were used to assay rescue of retinal signaling to the brain (Figure 6). Restoration of PLR implies successful transmission of photic information to the olivary pretectum of the brain. PLR of control *Lrat* ^−/−^ mice were ~3.5 log units less sensitive than heterozygous or WT animals (Figure 6I). Irradiance-response relations for PLR showed that both 9-*cis*-R-Ac and rAAV-*Lrat* treatments each increased *Lrat* ^−/−^ PLR sensitivity by ~2.5 log units (Figure 6I). Figure 6A–6D and Video S1 compare the PLRs of the same *Lrat* ^−/−^ mouse before and after 9-*cis*-R-Ac treatment, illustrating the significant gain in photosensitivity observed following treatment. Similarly, Figure 6E–6H and Video S2 show that rAAV-*Lrat* treatment conferred substantially increased sensitivity to light stimuli as compared with the control contralateral eye of the same animal. Similar improved PLR were recently obtained for *Rpe65* ^−/−^ mice treated with 9-*cis*-RAL [30].

### Complementarity of Viral and Pharmacological Rescue of *Lrat* ^−/−^ Mice

To determine if viral and pharmacologic treatments could be combined, we performed oral gavage on *Lrat* ^−/−^ mice previously rescued with rAAV-*Lrat*. Biochemical augmentation of visual pigment was observed directly, as the elution of 11-*cis*-RAL oximes (a result of AAV rescue) and 9-*cis*-RAL oximes (produced by oral gavage) were well separated on a HPLC column (Figure S3A–S3C). While ERG amplitudes of virally-rescued animals were below those seen in mice rescued by oral gavage, ERG responses in *Lrat* ^−/−^ mice treated with rAAV-*Lrat* and 9-*cis*-R-Ac further improved to reach the levels of 9-*cis*-R-Ac treated mice (Figure S3D and S3E).

## Discussion

Here, we demonstrate that inborn errors of metabolism leading to blinding diseases can be successfully treated by gene therapy or pharmacological intervention. Visual pigment formation, tissue morphology, and visual function as measured by single-cell recordings, ERG, and PLR were significantly improved after treating a mouse model of LCA due to deficiency in LRAT with intraocular injections of rAAV-*Lrat* or by oral gavage with 9-*cis*-R-Ac. Pharmacological interventions to treat some forms of retinal disease have been attempted in animal models of human blinding conditions, among them treatment of *rd1* mice with the Ca^2+^ channel blocker *D-cis-*diltiazem and other blockers that prevent rise of intracellular Ca^2+^ to toxic levels [31], and treatment of *rd1* mice with leukemia inhibitory factor, which is involved in the down-regulation of genes involved in synthesis and degradation of cGMP [32]. Other studies have used inhibitors of apaptosis, an approach applicable to a wide range of retinal disorders. Several studies suggest that intraocular injection of neurotrophic factors (e.g., brain-derived neurotrophic factor and ciliary neurotropic factor ) can protect the murine retina from light damage, or delay photoreceptor degeneration in animal models (for example [33]). In most cases, the beneficial effects of treatment last less than a month and require repeated administrations.

Dryja and colleagues showed that ROL supplementation slows the rate of photoreceptor degeneration caused by a T4M rhodopsin mutation in mice [34]. More recently, 9-*cis*-RAL application to *Rpe65* ^−/−^ mice resulted in formation of an active iso-rhodopsin and an improvement in the ERG of these animals for a time period of up to 6 mo after treatment [7,16]. The *Rpe65* mutation blocks the retinoid cycle by preventing the generation of 11-*cis*-RAL, the chromophore of visual pigments. Oral application of 9-*cis*-RAL circumvents this blockage. The blockade has also been rescued by rAVV-*Rpe65* gene transfer method in naturally *Rpe65*-deficient dogs [9]. *Rpe* ^−/−^ mice display enhanced esterification properties that could lead to trapping 9-*cis*-retinoids in the form of 9-*cis*-REs. Thus it was very important to examine if this pharmacological approach would be successful in mice lacking the key esterifying enzyme. Combining oral retinoid treatment (using next-generation retinoid pro-drugs) and viral rAAV-*Lrat* somatic gene therapy in *Lrat* ^−/−^ mice led to remarkable rescue of visual functions in these mice. We found that both methods increased ERG responses from ~5% of wild-type levels in *Lrat* ^−/−^ mice to ~50% of wild-type levels in treated *Lrat* ^−/−^ mice. Retinoid treatment led to increased levels of visual chromophore from undetectable levels to 600 pmoles per eye. The ROS dark current was restored to near-normal levels of 15 ± 1 pA after a single treatment with 9-*cis*-R-Ac, and the half-maximal flash strength after three treatments was 87 ± 6 photons/μm^2^. Correcting for the lowered 9-*cis*-RAL quantum efficiency, this is within a factor of 2 of WT mouse rod responses. Irradiance-response relations for PLR showed that both 9-*cis*-R-Ac and rAAV-*Lrat* treatments each increased *Lrat* ^−/−^ PLR sensitivity by ~2.5 log units.

### Rescue of Visual Functions by Pharmacological Treatment and Gene Transfer

Our previous findings described the *Lrat* ^−/−^ mouse as a model of LCA with pathological characteristics similar to those found in patients affected by mutations in the LRAT gene [11]. In the current study, we demonstrate that visual functions can be rescued in *Lrat* ^−/−^ mice, as measured by recovery of visual chromophore and pigment, and by single-cell and ERG responses. Successful restoration of retinotectal signaling, as measured by pupillary responses, was also achieved. Pharmacological treatment was successful in every experimental trial, with restoration of about half-maximal ERG responses compared with the WT mice. While full restitution of the ERG could not be obtained in *Lrat* ^−/−^ mice in the tested experimental conditions, our finding of nearly complete restitution of single cell responses suggests that remodeling of the neuronal retina in *Lrat* ^−/−^ may limit functional rescue [35]. Possibly, the ROS of *Lrat* ^−/−^ mice are shorter as a result of damage caused by phototransduction triggered by free opsin [27,28], a phenomenon reminiscent of ROS shrinkage under continuous stimulation by light (photostasis) or as a result of vitamin A deprivation [36–38]. However, the rod photoreceptors resist complete degeneration in *Lrat* ^−/−^ mice (even in 17-mo-old mice raised in a normal light cycle), while the cones undergo almost complete degeneration. In fact, the ROS in 17-mo-old mice are nearly as long as in 2-mo-old *Lrat* ^−/−^ mice (see Figure S2B). A similar observation has been made for *Rpe65*-deficent mice [39]. Multiple gavages during a 2-wk period led to morphological restoration once the ROS completed the 12-d cycle of phagocytosis and renewal [24]. The improved rescue after multiple gavages was confirmed not only by retinoid analysis by HPLC, but also by morphological analysis and electrophysiological tests. In single-cell recordings, the ~3-fold difference in sensitivity between WT ROS and ROS from multiply treated *Lrat* ^−/−^ mice could be accounted for by the discrepancy in the quantum yield of opsin loaded with 11-*cis*-RAL and 9-*cis*-RAL chromophores. These differences are small compared to the range of intensities over which vision operates [40].

Oral synthetic retinoid treatment was remarkably successful in rescuing the *Lrat* ^−/−^ phenotype because of the highly specific mechanism of ROL transport and retention [41]. ROL absorption is followed by entrapment of REs in the lipid droplets of hepatic stellate cells and the retinosome structures found in the RPE [12,42]. ROL is mobilized from the intestine or liver as ROL ester components of chylomicrons or as ROL complexed with ROL-binding protein and transthyretin or albumin [12,42–44]. *Cis*-retinoids employed in this study used the existing retinoid transport system and were efficiently delivered to the eye (see Figure 2C).

We have chosen 9-*cis*-retinoids in our studies because they display higher stability than 11-*cis*-retinoids in storage and handling and at the low pH of the stomach, and they require simpler chemical synthesis, yet they share many advantages of 11-*cis*-retinoids, including the efficient formation of highly sensitive pigments and a similar metabolic pathway of degradation and transport in mammals. Based on their chemical properties, pro-drug *cis*-REs appear to be a better alternative to RAL or ROL because of their stability and efficient metabolic transformation to active 9-*cis*-RAL in the eye. Interestingly, other pro-drugs, such as vitamin E, are also delivered as more hydrophilic succinate esters [45]. Such REs lower the hydrophobicity and non-specific diffusion of these lipid-soluble compounds within the membranes of the digestive tract. In the RPE, 9-*cis*-ROL is transiently trapped as REs (Table 1), which then undergo hydrolysis and oxidation to regenerate the active chromophore 9-*cis*-RAL (see Figure 1C). The absorption and transformation of *cis*-retinoids might be similar to that of all-*trans*-retinoids because of the multiple overlapping activities of the enzymes involved and their low specificity toward different retinoid isomers [41].

Viral treatment was also effective in restoring the normal retinoid cycle in the eye and rescuing visual function. The rescue peaked at 6 wk and then slowly decayed. The expression of *Lrat* was localized to the injection site, but significant rescue of visual pigment rhodopsin was observed (~50%). More eye-to-eye variability was observed with viral rescue than with pharmacologic rescue. Because of their small size and relatively large lenses, which occupy about 70% of the eye, subretinal injection surgery is more complicated for mouse eyes than it would be for larger eyes.

While direct comparison of rescue methods is difficult, it appears that both therapeutic methods provide efficient recovery of higher order visual responses, as tested by PLRs. The clear advantage of oral retinoid treatment is its ease of administration compared with the subretinal injections required for viral vectors. Its primary disadvantage is its potential for long-term systemic toxicity compared to vector targeting of LRAT to the RPE. The usual advantage of rAAV-delivered gene therapy over systemic drugs is the persistence of passenger gene expression after a single administration; however, in this case there was a gradual but distinct loss of delivered LRAT function after several months. This has not been observed in other systems (RPE65 dog and mouse studies) and may reflect an *Lrat* ^−/−^-specific effect on RPE viability. The slow and gradual decline in ERG amplitude in treated mice is also observed with age in *Lrat* ^+/+^ mice. Although the decline rate during the first 7 mo of life for *Lrat* ^+/+^ mice is slight (T. Maeda and KP, unpublished data) and is slower than in rAAV-treated mice, the direct comparison is not precise, as untreated retinas of *Lrat* ^−/−^ degenerate slowly, and the level of opsin expression appears to be slightly lower (unpublished data). Thus, the observed decline in ERG amplitudes could be a result of several different factors, including the stability of rAAV infection, retina remodeling in *Lrat* ^−/−^ mice [35], and shorter ROS structure in *Lrat* ^−/−^ mice (this study) and degeneration of cone photoreceptors (unpublished data). Importantly, 9-*cis*-R-Ac treatment appeared to improve rescue in virally treated animals, and resulted in reconstitution of both native 11-*cis-*retinoids and administered 9-*cis-*retinoids simultaneously.

### Insight into Retinoid Metabolism

The level of 9-*cis*-RAL is maintained in the eyes of 9-*cis*-R-Ac-treated *Lrat* ^−/−^ dark-adapted mice for over 4 mo. This finding suggests that the rod-RPE system efficiently recycles the chromophore of phagocytosed visual pigment. This phenomenon can be readily observed, because retinoids are only transiently stored in organs other than the eye in *Lrat* ^−/−^ mice. Approximately 10% of the ROS is renewed each day [46]. The entire length of the ROS is thus completely renewed in less than 2 wk. However, the 9-*cis*-RAL based pigment persists in the eye for 4 mo post-gavage at a ~50% level. This can be explained only by the recovery of 9-*cis*-chromophore from isorhodopsin phagocytosed by the RPE. Second, even though REs are transiently formed in the eye (Table 1), they do not support the retinoid cycle, suggesting that LRAT is a key enzyme in this process and that formation of esters is insufficient in the completion of the cycle. This observation suggests that localization of these esters is crucial for the proper function of the retinoid cycle. Third, we confirmed reports obtained by Fan et al. [23] that mice with an impaired retinoid cycle appear to spontaneously generate 9-*cis*-RAL that is then trapped by ROS containing almost exclusively free opsin.

### Leber Congenital Amaurosis and Potential Treatment

The major advantage of retinoid-based treatments of LCA resulting from the deficiency of LRAT is that these compounds are not stored in the liver for any prolonged time and are quickly oxidized and secreted. Retinoid absorption in mammals is an active process driven by esterification/hydrolysis cycles in the intestine and liver and in the RPE. Esterification is carried out mainly by LRAT [47], as evidenced by the fact that the absence of LRAT makes retinoids vulnerable to quick elimination from the body. In the absence of LRAT, the equilibrium between ROLs and REs is clearly shifted in favor of free ROL. Low levels of REs are present in mice deficient in LRAT expression (*Lrat* ^−/−^), but these esters are formed transiently as a possible consequence of acyl-CoA:retinol acyltransferase activity [25, 48].

An important finding is that the pharmacological treatment can be sustained multiple times, and that several low-dose treatments show cumulative effects. This treatment is possible at any age, as the morphological structure of the retina is to some degree preserved in both young and old mice. These data are reminiscent of results obtained following gene therapy of *RPE65*-deficient mice and dogs [7,9,12,16,49,50].

*Cis*- and all-*trans*-RALs are irreversibly oxidized to RAs by RAL dehydrogenase types 1, 2, 3, and 4 [51]. All-*trans*-RA and its 9-*cis*-isomer are important regulators of gene expression via nuclear receptors [52]. For any retinoid-based therapy there can be negative side effects resulting from potential production of RAs and their teratogenic effects. To prevent their accumulation, RAs are oxidized by CYP26A1, CYP26B1, and CYP26C1 to 4-hydroxy-RA, 4-oxo-RA, and 18-hydroxy-RA [53]. As demonstrated here, only a low and transient level of RAs is observed in the *Lrat* ^−/−^ mice gavaged with 9-*cis*-R-Ac, arguing that the potential side effects due to RA production are highly attenuated. More toxicological studies are needed before pharmacological treatment can be proposed for treatment of similar human conditions. It is possible that a combination of viral and pharmacological treatment could be the most efficient means of restoring vision in patients afflicted with LCA, for whom no treatment is currently available.

The rescue of PLRs in *Lrat* ^−/−^ demonstrates that treatment of the retinal defects results in ultimate improvement in signaling from eye to brain. PLRs are mediated by both inner retinal, non-visual photoreceptors [54] and rod and cone input. While measurement of the PLR in this study could not readily distinguish between rescue of inner and outer retinal photoreceptors, given the observed dramatic improvement of the ERG, these results suggest that rescued outer retinal function may be at least partly responsible for rescue of the PLR. Determination of the extent to which pharmacologic or somatic gene therapy rescue of *Lrat* ^−/−^ mice would allow restoration of actual visual function will require additional study. It is unknown at present to what extent mice lacking *Lrat* develop normal intraretinal circuitry, and it is similarly unknown to what extent these mice develop amblyopia from visual deprivation. The apparent visual improvement associated with rAAV rescue in dogs mutant in the *Rpe65* gene [9], however, does suggest that form vision may be restored in at least some forms of LCA.

Our findings establish that both chromophore supplementation and somatic gene therapy are effective in improving visual functions in the *Lrat* ^−/−^ mouse, an animal model of LCA that accurately replicates the pathology of the disease. The treatments are optimally effective in combination and if the chromophore supplementation is continued at low doses for a longer period of time. Applying 9-*cis*-R-Ac treatment to virally treated animals appeared to improve rescue, and resulted in reconstitution of both native 11-*cis-* and administered 9-*cis-* retinoids simultaneously. There is a great value in combined approaches, for several reasons. First, either one may prove to be more suitable for a specific age group of patients, thus offering effective treatment for a wider age range; second, a partial and regional rescue by rAVV, as observed in the *Rpe65*-deficient dog (unpublished data), could be augmented by retinoid treatment, reaching the whole retina; third, the rescue in rAVV-treated animals allows storage of 9-*cis*-REs that might be mobilized when needed (for example, high bleaching levels). Clinically, it is likely that pharmacologic and somatic gene therapeutic approaches, if successful, could be used in complementary fashion; for example, treatment of appropriate patients with oral retinoids could begin in infancy to avoid amblyopia, avoiding the difficulties associated with surgery in very young patients, while at older ages long-lasting drug-free treatment might be achieved by surgical introduction of viral vectors.

This work on the rescue of vision extends previous studies by Van Hooser et al. [7,16] in part by employing novel compounds with potentially better drug-like properties, and to the second LCA mouse model. In contrast to *Rpe65* ^−/−^ mice, *Lrat* ^−/−^ mice do not have significant capacity to store retinoids outside of the visual pigment; however, the pharmacological approach was highly successful. There have been reports documenting the potential adverse effects of high doses of retinoids [55]. During these and related studies of both acute and prolonged (up to 1 y) multiple dose treatments, no adverse effects were observed for mice of several genetic backgrounds. Number and size of litters, coat grooming, survival after gavage, post-natal development, and growth/weight curves were unaffected by treatment. *Lrat* ^−/−^ mice may be particularly resistant to potential toxicity of RE compounds, as lack of esterification of the retinoid agents actively leads to rapid removal through secretion and oxidation to oxo/hydroxo RA compounds. This reaction occurs not only in the eye but also in the liver, where LRAT is also normally expressed. The transient formation and accumulation of retinoids observed in these mice soon after treatment disappeared within a day in most cases. Only visual pigments had the capability to retain these retinoids for a long period of time, as shown in Figure 2H. These observations raise the hope that, after formal toxicological studies, these RE pro-drugs have the potential to be extended effectively to humans.

## Supporting Information

Figure S1 Chromatographic Separation of Retinoids Extracted from 16 *Lrat* ^−/−^ Mouse Eyes (A) Peak 1, syn-11-*cis*-RAL oxime; 2, undetermined compound with λ_max_ at 315 nm; 3, syn-all-*trans*-RAL oxime; 4, syn-9-*cis*-RAL oxime; 5, 13-*cis*-ROL; and 6, all-*trans*-ROL.
(B) Retinoids bound to immunoaffinity purified opsin from 40 untreated *Lrat* ^−/−^ mouse eyes (one-third of the sample was loaded on the HPLC column).
(125 KB PDF).

Figure S2 Levels of 9-*cis*-RAL in Mice of Different Ages Treated with 9-*cis*-R-Ac (A) 9-*cis*-RAL oxime levels in *Lrat* ^−/−^ mouse eyes following gavage with 20 μmol 9-*cis*-R-Ac at differing ages. The analysis is performed after 72 h of post-gavage dark adaptation (*n* ≥ 3).
(B) ROS thickness as a function of the retinal location from the optic nerve head (in mm). Four age-matched retinas of WT and *Lrat* ^−/−^ were analyzed both in inferior/superior and nasal/temporal orientations, and the data plotted with Microsoft Excel. The dotted lines represent data from 2-mo-old *Lrat* ^+/−^ and *Lrat* ^−/−^ mice as published earlier [10].
(60 KB PDF).

Figure S3 Retinoid Analysis and ERG of rAAV-*Lrat* Treated *Lrat* ^−/−^ Mice Augmented with 9-*cis*-R-Ac (A) *Lrat* ^−/−^ mouse treated with rAAV-*Lrat*. Inset, immunoaffinity-purified rhodopsin from the retina of *Lrat* ^−/−^ mice treated with rAAV-*Lrat* virus. E, corresponding fraction in elution.
(B) *Lrat* ^−/−^ mouse treated with 9-*cis*-R-Ac.
(C) *Lrat* ^−/−^ mouse treated with rAAV-*Lrat* and 9-*cis*-R-Ac. Peaks marked * represent the solvent change artifact. Inset, the chromatogram of retinoids from WT mouse.
(D) Scotopic single-flash ERG a-waves.
(E) Scotopic single-flash ERG b-waves (*n* ≥ 10, data shown with SEM).
(175 KB PDF).

Figure S4 Scatter Plot of a- and b-waves of ERGs Obtained from rAAV-*Lrat*-Treated and Control *Lrat* ^−/−^ Mice (A) ERG a-waves of 6- to 7-wk-old *Lrat* ^−/−^ control mice.
(B) ERG b-waves of 6- to 7- wk-old *Lrat* ^−/−^ control mice.
(C) ERG a-waves of 6- to 7- wk-old rAAV-*Lrat*-treated mice.
(D) ERG b-waves of 6- to 7- wk-old rAAV-*Lrat*-treated mice (*n* ≥ 39).
(129 KB PDF).

Video S1 Effect of 9-*cis*-R-Ac Treatment on the PLR of *Lrat* ^−/−^ Mice PLRs of an individual *Lrat* ^−/−^ mouse (left eye) recorded prior (control) and 72 h subsequent (9-*cis*-R-Ac) to three 5-μmol doses of 9-*cis*-R-Ac. Presence of light stimulus (30-s pulse of narrow bandpass 470 nm light, 1.38 × 10^14^ photons·cm^−2^·sec^−1^) is represented in the movie by a light bulb symbol.
(3.0 MB MOV).

Video S2 Effect of Intraocular Injection of rAAV-*Lrat* on the PLR of *Lrat* ^−/−^ Mice PLRs of an individual *Lrat* ^−/−^ mouse recorded from rAAV-*Lrat*-treated left eye (rAAV-*Lrat*) and non-treated right eye (control). Presence of light stimulus (30-s pulse of narrow bandpass 470 nm light, 4.79 × 10^13^ photons·cm^−2^·sec^−1^) is represented in the movie by a light bulb symbol.
(3.0 MB MOV).

### Accession Number

The GenBank (http://www.ncbi.nlm.nih.gov/) accession number of Chromosome 11q13 is AF139813.

**Patient Summary**

**Background**

Some causes of blindness are inherited. Leber congenital amaurosis is one inherited disease that causes degeneration and loss of activity of the retina—the tissue at the back of the eye. Hence, babies have a severe loss of vision at birth as well as roving eye movements (nystagmus), deep-set eyes, and sensitivity to bright light. One cause of Leber congenital amaurosis is loss of an enzyme called lecithin:retinol acyl transferase (LRAT), which is required for regeneration of a pigment necessary for the eye to detect light. Currently there is no treatment for this condition.

**Why Was This Study Done?**

There are animal models of this disease, and previous work has suggested that there are two possible ways of treating this condition. One is by giving by mouth synthetic pigments similar to those in the eye. Another is by gene therapy with viruses that replace the abnormal gene with normal copies. The authors wanted to test these methods in a mouse model, and see if the two approaches worked well together.

**What Did the Researchers Do and Find?**

They treated mice that had a genetic defect mimicking the human condition by placing a virus carrying the normal gene directly into their eyes. They also gave the mice pro-drugs (compounds that are turned into the active drug inside the body) by mouth, which bypassed the missing step in the regeneration of the retinal compound. The authors found that they got the best results either using individual methods or when they tried both approaches together. They could show that the mice had the electrical impulses that are a sign that the eye is working correctly, and in addition, their pupils responded to light.

**What Do These Findings Mean?**

These results are an early step to making these treatments available to patients. Obviously, they would only help patients who had this particular genetic defect, and possible defects in genes from this same metabolic pathway. Before these treatments were to be used, many other questions would need to be answered, including whether these oral compounds might be toxic if given repeatedly—as they would need to be. Also, acceptable methods of placing the gene therapy into human eyes would need to be found.

**Where Can I Get More Information Online?**

The Foundation Fighting Blindness funds research on retinal degenerative diseases, and has a number of pages of information for patients and families:

http://www.blindness.org/

Contact a Family is a UK charity that has information on specific conditions and rare disorders:

http://www.cafamily.org.uk

MedlinePlus also has a series of pages on retinal disorders:

http://www.nlm.nih.gov/medlineplus/retinaldisorders.html

## Abbreviations

AAV: adeno-associated virus
EM: electron microscopy
ERG: electroretinogram
LCA: Leber congenital amaurosis
LRAT: lecithin:retinol acyl transferase
PLR: pupillary light response
RA: retinoic acid
R-Ac: retinyl acetate
RAL: retinal
RE: retinyl ester
ROL: retinol
ROS: rod outer segment
RPE: retinal pigment epithelium
R-Su: retinyl succinate
SD: standard deviation
SEM: standard error of the mean
WT: wild-type

## References

[pmed-0020333-b01] Rattner A, Sun H, Nathans J (1999). Molecular genetics of human retinal disease. Annu Rev Genet.

[pmed-0020333-b02] Baehr W, Wu SM, Bird AC, Palczewski K (2003). The retinoid cycle and retina disease. Vision Res.

[pmed-0020333-b03] Jacobson SG, Cideciyan AV, Regunath G, Rodriguez FJ, Vandenburgh K (1995). Night blindness in Sorsby's fundus dystrophy reversed by vitamin A. Nat Genet.

[pmed-0020333-b04] Lotery AJ, Namperumalsamy P, Jacobson SG, Weleber RG, Fishman GA (2000). Mutation analysis of 3 genes in patients with Leber congenital amaurosis. Arch Ophthalmol.

[pmed-0020333-b05] Cremers FP, van den Hurk JA, den Hollander AI (2002). Molecular genetics of Leber congenital amaurosis. Hum Mol Genet.

[pmed-0020333-b06] Redmond TM, Yu S, Lee E, Bok D, Hamasaki D (1998). Rpe65 is necessary for production of 11-*cis*-vitamin A in the retinal visual cycle. Nat Genet.

[pmed-0020333-b07] Van Hooser JP, Aleman TS, He YG, Cideciyan AV, Kuksa V (2000). Rapid restoration of visual pigment and function with oral retinoid in a mouse model of childhood blindness. Proc Natl Acad Sci U S A.

[pmed-0020333-b08] Campochiaro PA (2002). Gene therapy for retinal and choroidal diseases. Expert Opin Biol Ther.

[pmed-0020333-b09] Acland GM, Aguirre GD, Ray J, Zhang Q, Aleman TS (2001). Gene therapy restores vision in a canine model of childhood blindness. Nat Genet.

[pmed-0020333-b10] Batten ML, Imanishi Y, Maeda T, Tu DC, Moise AR (2004). Lecithin-retinol acyltransferase is essential for accumulation of all-trans-retinyl esters in the eye and in the liver. J Biol Chem.

[pmed-0020333-b11] Thompson DA, Li Y, McHenry CL, Carlson TJ, Ding X (2001). Mutations in the gene encoding lecithin retinol acyltransferase are associated with early-onset severe retinal dystrophy. Nat Genet.

[pmed-0020333-b12] Imanishi Y, Batten ML, Piston DW, Baehr W, Palczewski K (2004). Noninvasive two-photon imaging reveals retinyl ester storage structures in the eye. J Cell Biol.

[pmed-0020333-b13] Zolotukhin S, Potter M, Hauswirth WW, Guy J, Muzyczka N (1996). A “humanized” green fluorescent protein cDNA adapted for high-level expression in mammalian cells. J Virol.

[pmed-0020333-b14] Esumi N, Oshima Y, Li Y, Campochiaro PA, Zack DJ (2004). Analysis of the VMD2 promoter and implication of E-box binding factors in its regulation. J Biol Chem.

[pmed-0020333-b15] Maeda T, Van Hooser JP, Driessen CA, Filipek S, Janssen JJ (2003). Evaluation of the role of the retinal G protein-coupled receptor (RGR) in the vertebrate retina in vivo. J Neurochem.

[pmed-0020333-b16] Van Hooser JP, Liang Y, Maeda T, Kuksa V, Jang GF (2002). Recovery of visual functions in a mouse model of Leber congenital amaurosis. J Biol Chem.

[pmed-0020333-b17] Palczewski K, Van Hooser JP, Garwin GG, Chen J, Liou GI (1999). Kinetics of visual pigment regeneration in excised mouse eyes and in mice with a targeted disruption of the gene encoding interphotoreceptor retinoid-binding protein or arrestin. Biochemistry.

[pmed-0020333-b18] Zhu L, Jang GF, Jastrzebska B, Filipek S, Pearce-Kelling SE (2004). A naturally occurring mutation of the opsin gene (T4R) in dogs affects glycosylation and stability of the G protein-coupled receptor. J Biol Chem.

[pmed-0020333-b19] Sampath AP, Rieke F (2004). Selective transmission of single photon responses by saturation at the rod-to-rod bipolar synapse. Neuron.

[pmed-0020333-b20] Kane MA, Chen N, Sparks S, Napoli JL (2005). Quantification of endogenous retinoic acid in limited biological samples by LC/MS/MS. Biochem J.

[pmed-0020333-b21] Klvanova J, Brtko J (2002). Selected retinoids: Determination by isocratic normal-phase HPLC. Endocr Regul.

[pmed-0020333-b22] Haeseleer F, Jang GF, Imanishi Y, Driessen CA, Matsumura M (2002). Dual-substrate specificity short chain retinol dehydrogenases from the vertebrate retina. J Biol Chem.

[pmed-0020333-b23] Fan J, Rohrer B, Moiseyev G, Ma JX, Crouch RK (2003). Isorhodopsin rather than rhodopsin mediates rod function in RPE65 knock-out mice. Proc Natl Acad Sci U S A.

[pmed-0020333-b24] Bok D (1985). Retinal photoreceptor-pigment epithelium interactions. Friedenwald lecture. Invest Ophthalmol Vis Sci.

[pmed-0020333-b25] Yen CL, Monetti M, Burri BJ, Farese RV (2005). The triacylglycerol synthesis enzyme DGAT1 also catalyzes the synthesis of diacylglycerols, waxes, and retinyl esters. J Lipid Res.

[pmed-0020333-b26] Dowling JE (1960). Chemistry of visual adaptation in the rat. Nature.

[pmed-0020333-b27] Palczewski K, Saari JC (1997). Activation and inactivation steps in the visual transduction pathway. Curr Opin Neurobiol.

[pmed-0020333-b28] Woodruff ML, Wang Z, Chung HY, Redmond TM, Fain GL (2003). Spontaneous activity of opsin apoprotein is a cause of Leber congenital amaurosis. Nat Genet.

[pmed-0020333-b29] Saari JC, Garwin GG, Van Hooser JP, Palczewski K (1998). Reduction of all-trans-retinal limits regeneration of visual pigment in mice. Vision Res.

[pmed-0020333-b30] Fu Y, Zhong H, Wang M-HH, Luo D-G, Liao HW (2005). Intrinsically photosensitive retinal ganglion cells detect light with a vitamin A-based photopigment, melanopsin. Proc Natl Acad Sci U S A.

[pmed-0020333-b31] Frasson M, Sahel JA, Fabre M, Simonutti M, Dreyfus H (1999). Retinitis pigmentosa: Rod photoreceptor rescue by a calcium-channel blocker in the rd mouse. Nat Med.

[pmed-0020333-b32] LaVail MM, Yasumura D, Matthes MT, Lau-Villacorta C, Unoki K (1998). Protection of mouse photoreceptors by survival factors in retinal degenerations. Invest Ophthalmol Vis Sci.

[pmed-0020333-b33] Okoye G, Zimmer J, Sung J, Gehlbach P, Deering T (2003). Increased expression of brain-derived neurotrophic factor preserves retinal function and slows cell death from rhodopsin mutation or oxidative damage. J Neurosci.

[pmed-0020333-b34] Li T, Sandberg MA, Pawlyk BS, Rosner B, Hayes KC (1998). Effect of vitamin A supplementation on rhodopsin mutants threonine-17 –> methionine and proline-347 –> serine in transgenic mice and in cell cultures. Proc Natl Acad Sci U S A.

[pmed-0020333-b35] Jones BW, Watt CB, Frederick JM, Baehr W, Chen CK (2003). Retinal remodeling triggered by photoreceptor degenerations. J Comp Neurol.

[pmed-0020333-b36] Penn JS, Williams TP (1986). Photostasis: Regulation of daily photon-catch by rat retinas in response to various cyclic illuminances. Exp Eye Res.

[pmed-0020333-b37] Hofmann KP, Schleicher A, Emeis D, Reichert J (1981). Light-induced axial and radial shrinkage effects and changes of the refractive index in isolated bovine rod outer segments and disc vesicles: Physical analysis of near-infrared scattering changes. Biophys Struct Mech.

[pmed-0020333-b38] Naash MI, LaVail MM, Anderson RE (1989). Factors affecting the susceptibility of the retina to light damage. Prog Clin Biol Res.

[pmed-0020333-b39] Znoiko SL, Rohrer B, Lu K, Lohr HR, Crouch RK (2005). Downregulation of cone-specific gene expression and degeneration of cone photoreceptors in the *rpe65*
^−/−^ mouse at early ages. Invest Ophthalmol Vis Sci.

[pmed-0020333-b40] Baylor DA (1987). Photoreceptor signals and vision. Proctor lecture. Invest Ophthalmol Vis Sci.

[pmed-0020333-b41] McBee JK, Palczewski K, Baehr W, Pepperberg DR (2001). Confronting complexity: The interlink of phototransduction and retinoid metabolism in the vertebrate retina. Prog Retin Eye Res.

[pmed-0020333-b42] Imanishi Y, Gerke V, Palczewski K (2004). Retinosomes: New insights into intracellular managing of hydrophobic substances in lipid bodies. J Cell Biol.

[pmed-0020333-b43] Vogel S, Piantedosi R, O'Byrne SM, Kako Y, Quadro L (2002). Retinol-binding protein-deficient mice: Biochemical basis for impaired vision. Biochemistry.

[pmed-0020333-b44] Harrison EH (2005). Mechanisms of digestion and absorption of dietary vitamin A. Annu Rev Nutr.

[pmed-0020333-b45] Kline K, Yu W, Sanders BG (2004). Vitamin E and breast cancer. J Nutr.

[pmed-0020333-b46] LaVail MM (1976). Rod outer segment disk shedding in rat retina: Relationship to cyclic lighting. Science.

[pmed-0020333-b47] Ruiz A, Winston A, Lim YH, Gilbert BA, Rando RR (1999). Molecular and biochemical characterization of lecithin retinol acyltransferase. J Biol Chem.

[pmed-0020333-b48] O'Byrne SM, Wongsiriroj N, Libien JM, Vogel S, Goldberg IJ (2005). Retinoid absorption and storage is impaired in mice lacking lecithin: Retinol acyltransferase (LRAT). J Biol Chem.

[pmed-0020333-b49] Liang Y, Fotiadis D, Filipek S, Saperstein DA, Palczewski K (2003). Organization of the G protein-coupled receptors rhodopsin and opsin in native membranes. J Biol Chem.

[pmed-0020333-b50] Aleman TS, Jacobson SG, Chico JD, Scott ML, Cheung AY (2004). Impairment of the transient pupillary light reflex in *Rpe65*
^−/−^ mice and humans with Leber congenital amaurosis. Invest Ophthalmol Vis Sci.

[pmed-0020333-b51] Zhao D, McCaffery P, Ivins KJ, Neve RL, Hogan P (1996). Molecular identification of a major retinoic-acid-synthesizing enzyme, a retinaldehyde-specific dehydrogenase. Eur J Biochem.

[pmed-0020333-b52] Chambon P (1996). A decade of molecular biology of retinoic acid receptors. Faseb J.

[pmed-0020333-b53] Fujii H, Sato T, Kaneko S, Gotoh O, Fujii-Kuriyama Y (1997). Metabolic inactivation of retinoic acid by a novel P450 differentially expressed in developing mouse embryos. EMBO J.

[pmed-0020333-b54] Berson DM (2003). Strange vision: Ganglion cells as circadian photoreceptors. Trends Neurosci.

[pmed-0020333-b55] Collins M.D, Mao G.E (1999). Teratology of retinoids. Annu Rev Pharmacol Toxicol.

