# Supplementary material for: Pharmacological and rAAV Gene Therapy Rescue of Visual Functions in a Blind Mouse Model of Leber Congenital Amaurosis
Source: PLoS Med. 2005 Nov 1;2(11):e333. doi: 10.1371/journal.pmed.0020333 (PMC1274279; doi:10.1371/journal.pmed.0020333)
Supplement: Supplementary file 1 [file pmed.0020333.sg001.pdf]

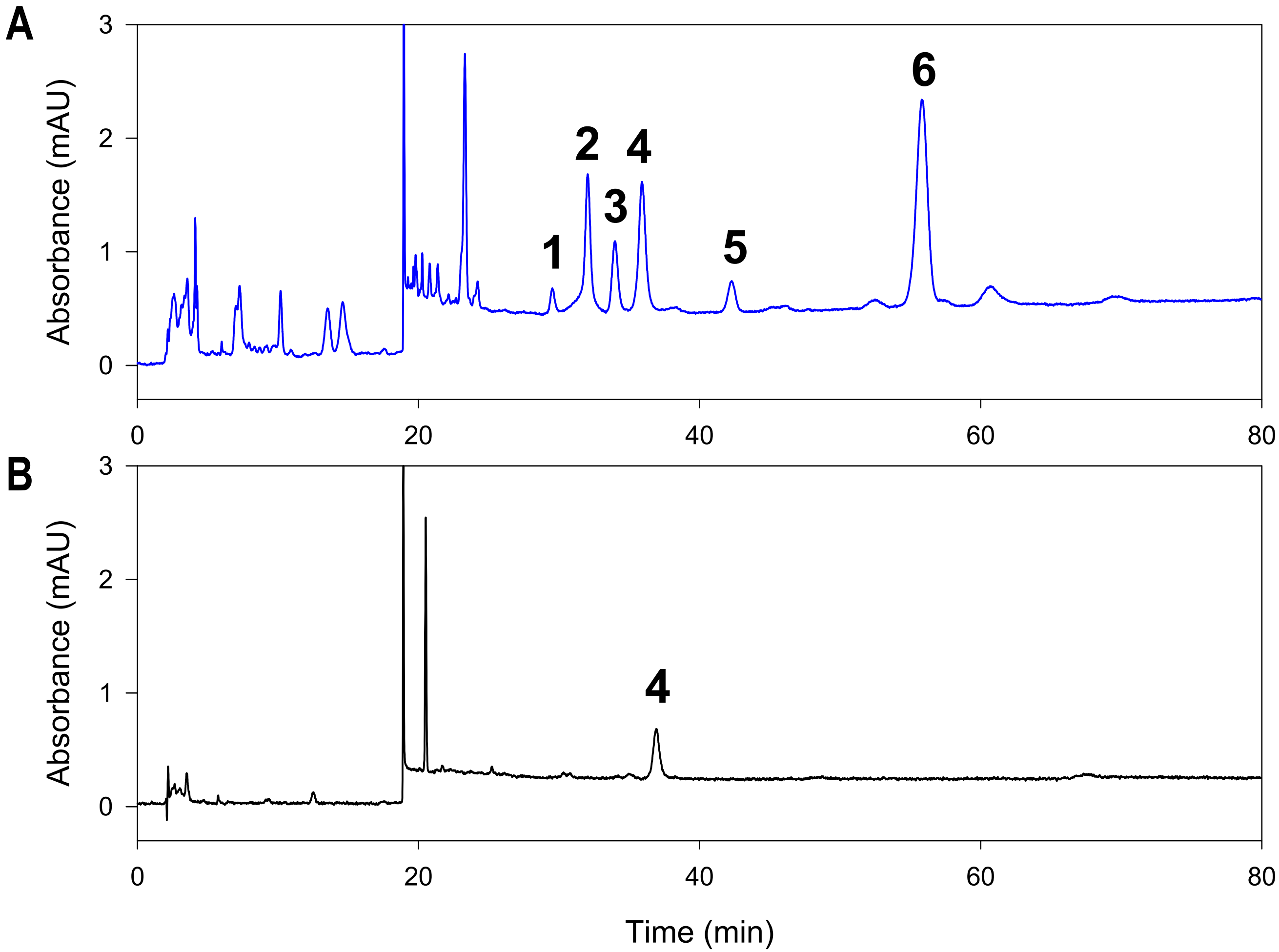

1.	*syn*-11-*cis*-retinal oxime
2.	undetermined compound with λ_{max} at 315 nm
3.	*syn*-all-*trans*-retinal oxime
4.	*syn*-9-*cis*-retinal oxime
5.	13-*cis*-retinol
6.	all-*trans*-retinol

Figure 1, supplement
