# Supplementary material for: Pharmacological and rAAV Gene Therapy Rescue of Visual Functions in a Blind Mouse Model of Leber Congenital Amaurosis
Source: PLoS Med. 2005 Nov 1;2(11):e333. doi: 10.1371/journal.pmed.0020333 (PMC1274279; doi:10.1371/journal.pmed.0020333)
Supplement: Supplementary file 3 [file pmed.0020333.sg003.pdf]

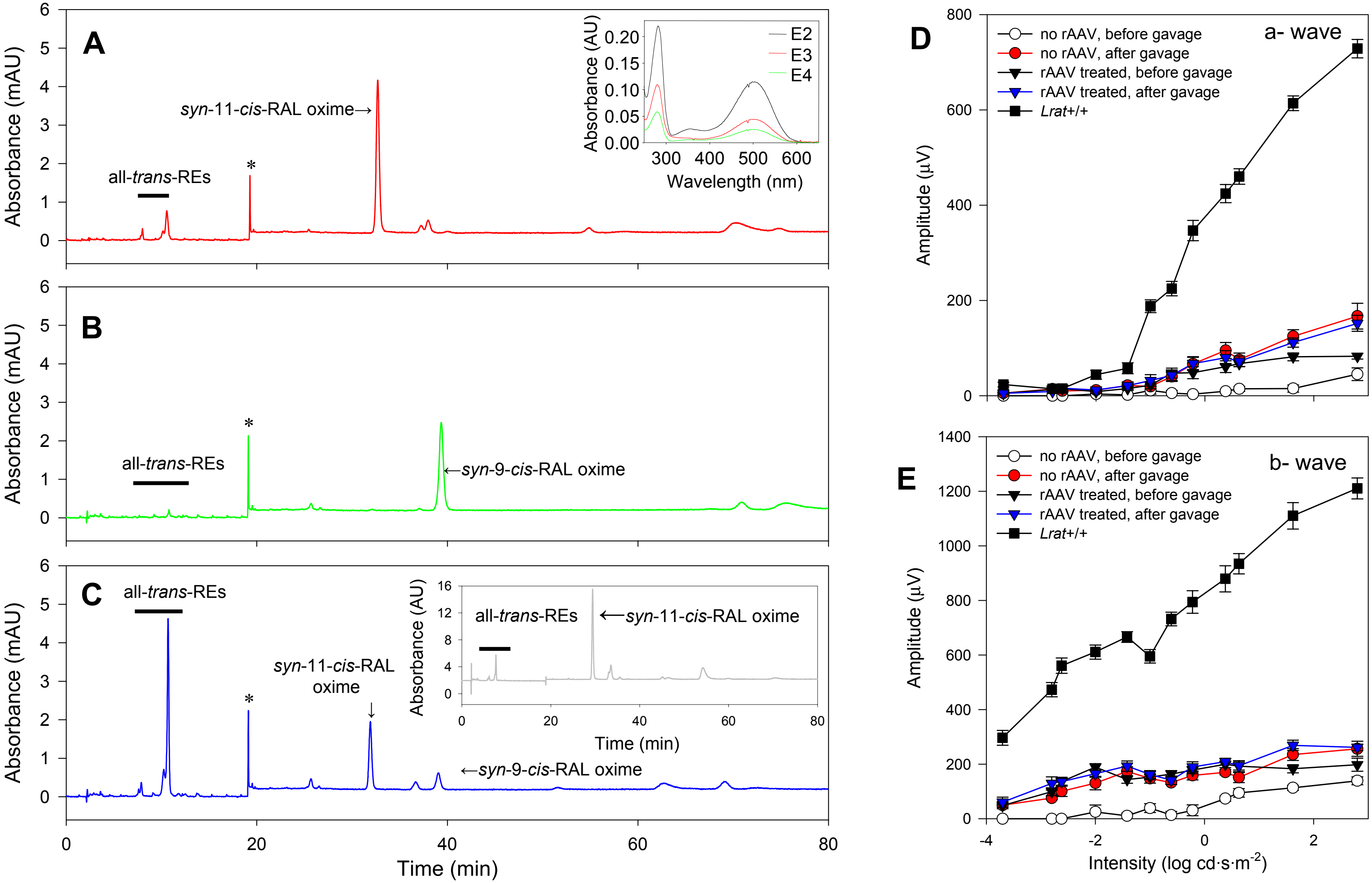

Figure 3, supplement
